# Smart molecular probes with controllable photophysical property for smart medicine

**DOI:** 10.1002/smo.20240033

**Published:** 2024-09-04

**Authors:** Xiaoying Kang, Zekun Du, Shuxuan Yang, Mengyun Liang, Qian Liu, Ji Qi

**Affiliations:** ^1^ State Key Laboratory of Medicinal Chemical Biology Key Laboratory of Bioactive Materials Ministry of Education, Frontiers Science Center for Cell Responses, and College of Life Sciences Nankai University Tianjin China; ^2^ Department of Urology Tianjin First Central Hospital Tianjin China; ^3^ Nankai International Advanced Research Institute Shenzhen China

**Keywords:** activatable, aggregate, molecular probe, photophysical property, theranostics

## Abstract

Precision medicine calls for advanced theranostics that integrate controllable diagnostic and therapeutic capabilities into one platform for disease treatment in the early stage. Phototheranostics such as fluorescence imaging (FLI), photoacoustic imaging (PAI), photodynamic therapy (PDT), and photothermal therapy (PTT) have attracted considerable attention in recent years, which mainly employ different excited‐state energy dissipation pathways of a chromophore. According to the Jablonski diagram, FLI is related to the radiative process, PAI and PTT are derived from the nonradiative thermal deactivation, and PDT originates from the triplet state energy, in which these processes are usually competitive. Therefore, it is critically important to precisely tune the photophysical energy transformation processes to realize certain diagnosis and treatment properties in optimal state for boosting biomedical applications. Currently, there are mainly two strategies including chemical structure and aggregate behavior changes that relate to the regulation of excited state energy dissipation. In this review, we will discuss the recent advances of smart molecular probes that the photophysical properties can be regulated by external triggers and their applications in biomedical fields. We will summarize the development of activatable phototheranostic molecular probes in response to stimuli such as reactive oxygen species, pH, light, hypoxia, enzyme and gas. The assembly and disassembly of molecular aggregates that greatly affect the photophysical energy transformation processes will also be highlighted. This review aims to provide valuable insights into the development of more accurate diagnostic and therapeutic systems, thereby advancing the emerging field of smart medicine.

## Graphical abstract

This review summarizes the recent advances in regulating the photophysical energy transformation processes of smart molecular probes through chemical structure and aggregate behavior changes. The development of activatable phototheranostic molecular probes triggered by stimuli such as reactive oxygen species, pH, light, hypoxia, enzyme, and gas as well as their biomedical applications have been discussed, rendering great promise for smart medicine.

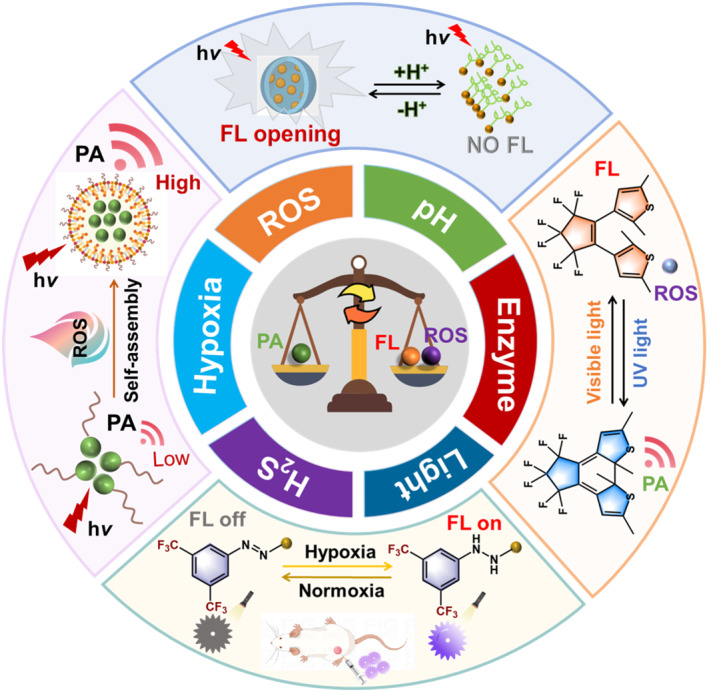

## 1 INTRODUCTION

Precision medicine calls for advanced theranostics that integrate controllable diagnostic and therapeutic capabilities into one platform for early disease treatment.[^1^, ^2^, ^3^, ^4^, ^5^] Conventional clinical diagnostic methods, including ultrasound, computed tomography (CT),[^6^, ^7^, ^8^, ^9^] and magnetic resonance imaging (MRI),[^10^, ^11^] suffer from low sensitivity and are unable to detect early‐stage diseases, preventing timely treatment. In recent years, optical imaging techniques such as fluorescence imaging (FLI),[^12^, ^13^, ^14^, ^15^, ^16^] and photoacoustic imaging (PAI),[^17^, ^18^, ^19^] have garnered significant research interest. FLI has emerged as a powerful tool for scientific research and medical diagnosis due to its high sensitivity, excellent spatiotemporal resolution, non‐invasiveness, and low cost.[^20^, ^21^, ^22^] PAI, which relies on the generation of varying ultrasound amplitudes and frequencies within the body through photothermal conversion to produce acoustic images, offers great penetration depth and high signal‐to‐noise ratio.[^23^, ^24^, ^25^, ^26^, ^27^, ^28^] However, the limitation of using a single imaging modality lies in its inability to simultaneously fulfill the requirements of non‐invasiveness, high sensitivity, specificity, spatial resolution, and penetration depth.[^29^, ^30^] Additionally, the integration of simultaneous diagnosis and treatment remains challenging in clinical practice, hindering the efficiency and accuracy of disease management to some extent.[^31^, ^32^, ^33^] Currently, the primary methods for treating cancers include surgery, chemotherapy, and radiotherapy.[^34^, ^35^, ^36^, ^37^, ^38^] When necessary, targeted therapy and biological therapy may also be combined. However, these treatments often involve invasive surgery, unpredictable toxicity, and drug resistance, resulting in a high risk of postoperative recurrence and difficulty in achieving complete cure. Therefore, more efficient treatment strategies are highly desirable for improving the therapeutic outcomes.

Phototherapy has emerged as a promising non‐invasive anti‐cancer approach, primarily encompassing photodynamic therapy (PDT) and photothermal therapy (PTT). PDT involves the light‐triggered excitation of a photosensitizer to a singlet excited state, which then transitions to a triplet excited state through intersystem crossing (ISC). The electron in the triplet state reacts with surrounding oxygen and water to produce highly toxic reactive oxygen species (ROS), leading to tumor vascular arrest and cancer cell death. However, the effectiveness of PDT is somewhat limited by the hypoxic nature of the tumor microenvironment, which necessitates the presence of oxygen.[^39^, ^40^, ^41^, ^42^, ^43^, ^44^, ^45^, ^46^, ^47^, ^48^, ^49^, ^50^, ^51^] On the other hand, PTT can generate high temperatures to destroy malignant tumor cells, even in hypoxic environments, offering an effective alternative to PDT.[^52^, ^53^, ^54^, ^55^, ^56^, ^57^, ^58^, ^59^, ^60^, ^61^, ^62^, ^63^, ^64^, ^65^] According to Jablonski diagram, FLI, PAI, PTT, and PDT are competitive processes, each representing different energy dissipation pathways for a single chromophore.[^66^, ^67^] Consequently, optimizing the performance of various phototheranostic properties is highly desirable but really challenging. To address this, modulating the molecular structure to fine‐tune the photophysical energy transformation processes is crucial for achieving the desired biomedical application performance. Additionally, the controllable aggregation and/or disaggregation of organic dyes significantly influence their photophysical properties. For instance, phenomena such as aggregation‐caused quenching (ACQ) and aggregation‐induced emission (AIE) are known to affect the phototheranostic properties in the aggregate state.[^68^, ^69^, ^70^, ^71^, ^72^, ^73^, ^74^] Therefore, altering molecular aggregation states provides an opportunity to tune photophysical energy transformation.[^66^, ^75^, ^76^, ^77^, ^78^, ^79^, ^80^, ^81^, ^82^, ^83^, ^84^, ^85^] Modulation of the photophysical properties of molecular probes in response to specific stimuli represents a novel design strategy for achieving smart diagnostic and therapeutic solutions.

Currently, most clinically used photoagents exhibit ‘permanent’ photoactivity across PDT, PTT, FLI, and PAI, resulting in inevitable side effects and suboptimal diagnostic accuracy. However, the overexpression of various active substances in tumor sites has enabled the development of activatable organic probes with on‐demand changes in photophysical properties. This advancement is critical for designing multifunctional nanomedicines and enhancing their biomedical applications.[^86^, ^87^, ^88^, ^89^, ^90^, ^91^] Many diseases, including cancer, inflammation, vascular diseases, and neurodegenerative disorders,[^92^, ^93^, ^94^] possess the overexpression of pathological biomarkers at the lesion site or exhibit microenvironmental changes such as pH,[^95^, ^96^, ^97^, ^98^, ^99^, ^100^] hypoxia,[^101^, ^102^, ^103^] and enzyme levels.[^104^, ^105^, ^106^] Therefore, the specific detection and diagnosis of these endogenous biomarkers at the early stages facilitate precise diagnosis and timely intervention of disease, which is of great significance for clinical applications. In recent years, the development of activatable organic molecular probes has garnered significant attention. The most common biomarkers include 1. ROS: These byproducts of aerobic metabolism are a group of oxygen‐containing reactive substances. The primary source of ROS is the substrate end of the respiratory chain in the inner membrane of mitochondria. During aerobic respiration, electron transfer chain complexes in mitochondria transfer electrons to O_2_, partially reducing it to forms such as superoxide (O_2_^−^),[^107^, ^108^] hydrogen peroxide (H_2_O_2_),[^109^, ^110^] singlet oxygen (^1^O_2_), hydroxyl radicals (^•^OH), and peroxyl radicals (ROO^•^).[^111^, ^112^, ^113^, ^114^, ^115^] 2. Reactive nitrogen species (RNS): These species result from interactions between nitric oxide (NO) and other compounds, producing highly oxidative free radicals and nitro compounds, including peroxynitrite (ONOO^−^) and its protonated form HOONO, S‐nitrosothiols (RSNO), and S‐nitrosoglutathione (GSNO).[^116^, ^117^, ^118^, ^119^, ^120^, ^121^, ^122^, ^123^] 3. Reactive carbonyl species (RCS): This family of small molecules contains one or more carbonyl groups and is mainly produced by the metabolism of carbohydrates, lipids, proteins, and amino acids. Key RCS include carbon monoxide (CO), formaldehyde (FA), glyoxal (GO), acrolein, and glucosone.[^124^, ^125^, ^126^] These responsive molecules enable specific reactions at disease sites by modulating the spectral response region, altering imaging modalities, and improving sensitivity, thereby reducing damage to normal tissues.

Except for the changes of molecular structures upon specific triggers, the regulation of molecular self‐assembly also offers a new avenue for controlling the photophysical properties and enhancing biomedical performance. By incorporating stimuli‐responsive groups into organic molecules, nano‐assembly systems can respond to specific triggers, thereby achieving controllable diagnostic and therapeutic performance.^[127]^ In 2001, Tang and co‐workers first introduced the concept of AIE, which refers to a new class of optical materials that are weakly or non‐emissive in dilute solutions but emit brightly in aggregated form.[^128^, ^129^, ^130^, ^131^, ^132^] For AIE luminogens (AIEgens), the excited state energy is dissipated through intensive intramolecular motion in solution via nonradiative decay. However, in the aggregate state, the molecular motion is restricted, closing the nonradiative pathway and opening the radiative process. Since small organic dyes are typically hydrophobic, they usually self‐assemble into nanoparticles to enhance their water solubility and biocompatibility. Consequently, constructing responsive self‐assembled nanosystems could regulate the photophysical properties and improve the phototheranostic performance of molecular probes.^[30]^

**FIGURE 1:**
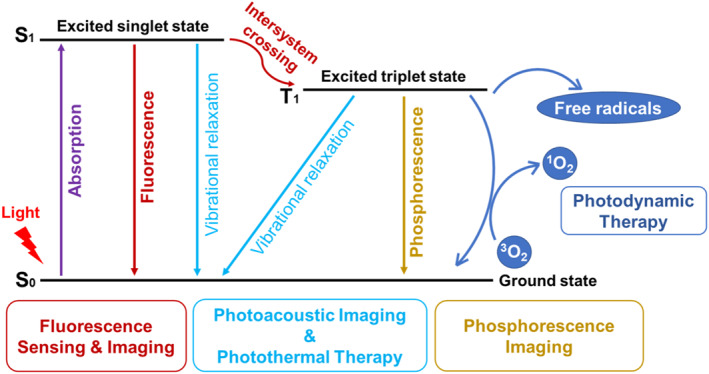
Jablonski diagram and its important role in designing phototheranostic agents for different applications.

**FIGURE 2:**
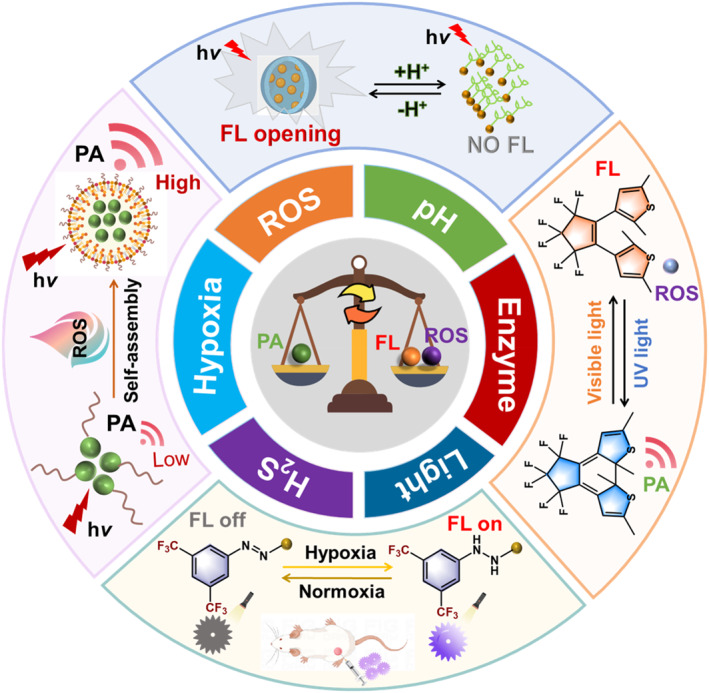
The modulation of photophysical energy transformation processes in organic molecular probes in response to specific stimuli for smart medicine.

Phototheranostic properties are closely related to the photophysical energy transformation processes (Figure 1), so it is critical to tune these processes to obtain optimal performance. The two main strategies include the modulation of chemical structure and aggregate behavior. There are multiple pathological characteristics that can be employed to trigger the transformation, therefore boosting the biomedical applications. In this review, we focus on the modulation of photophysical properties using various smart molecular probes and their applications in biomedical fields (Figure 2). We will discuss the recent advances in activatable FLI, PAI, PDT, and PTT in response to stimuli such as ROS, pH, light, hypoxia, enzyme, and gas as well as the assembly and disassembly of molecular aggregates. This review aims to provide valuable insights for the development of more accurate diagnostic and therapeutic systems, thereby advancing the field of smart medicine.

## 2 ROS‐RESPONSIVE MOLECULAR PROBES

**FIGURE 3:**
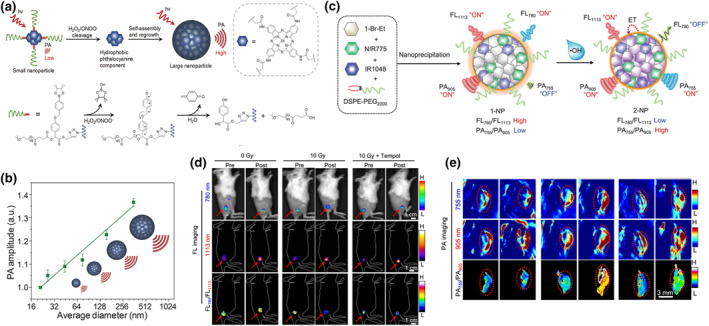
(a) Design mechanism of the self‐assembly polymer probe (PCBP) for PA imaging of ROS. (b) Average diameters and PA amplitudes of PCBP with the treatment of ROS in PBS (pH = 7.4). Reproduced with permission.^[139]^ Copyright 2017, WILEY‐VCH Verlag GmbH & Co.KGaA, Weinheim. (c) Design mechanism of 1‐NP and its transformation in response to ^•^OH. (d) Ratiometric FLI and (e) PAI of 4T1 tumors following indicated treatments. Reproduced with permission.^[140]^ Copyright 2018, The Author(s).

ROS are specific biomarkers associated with numerous pathological processes, making ROS‐responsive probes a focal point of recent research.^[133]^ The ability to tune the molecular structure before and after ROS stimuli can significantly alter its photophysical properties and biomedical performance.[^134^, ^135^, ^136^, ^137^, ^138^] To enhance the PA signal of molecular probes at disease sites, Pu and co‐workers designed and synthesized a ROS‐responsive semiconductor polymer probe capable of self‐assembly (Figure 3a).^[139]^ As the molecular structure changed, the PA signal increased, making it suitable for in vivo imaging of ROS. Zinc tetra(2‐(1‐(3‐amino‐3‐oxopropyl)‐1*H*‐1,2,3‐triazol‐4‐yl)methoxy)‐2‐oxo‐1‐(4‐(4‐(4,4,5,5‐tetramethyl‐1,3,2‐dioxaborolan‐2‐yl)benzoyloxy)phenyl)ethyl methoxypoly(ethylene glycol) succinate phthalocyanine (PCBP) contained two functional parts: phthalocyanine for PAI and a poly(ethylene glycol) (PEG) chain to enhance water solubility. Finally, a phenylboronic acid pinacol ester group was conjugated to provide responsiveness to H_2_O_2_/ONOO^−^. Upon entering the tumor site, the arylboronic ester group was oxidized by H_2_O_2_ and ONOO^−^, cleaving the hydrophilic PEG from PCBP and releasing hydrophobic phthalocyanine. Owing to the hydrophobic interactions and increased π‐π stacking, the phthalocyanine fragments would self‐assemble into larger nanoparticles. Previous researches have shown that larger nanoparticles have a stronger heat transfer ability due to the increased internal heat transfer rate, resulting in stronger PA signals. This was subsequently verified in the experiment, where both the increased concentration of PCBP and the diameter of self‐assembled polymers contributed to the enhancement of PA signals (Figure 3b). To test the in vivo performance, a subcutaneous 4T1 xenograft tumor model was established. To further increase the ROS level at the tumor site, _*D*,*L*_‐buthionine‐(S,R)‐sulfoximine (BSO) was used to reduce the glutathione levels. In the BSO group, the PA signal reached a maximum at 8 h post PCBP injection, and remained clear even after 30 h. The particles exhibited good targeting ability with PA signals enriched at the tumor site. This work demonstrates that the self‐assembly of large nanoparticles, triggered by a precise response at the disease site, can enhance PA signals, offering new strategies for achieving smart diagnostics for other diseases.

In order to enable non‐invasive monitoring of ^•^OH generated during tumor radiation therapy (RT) or ferroptosis, Ye et al. developed a ^•^OH‐responsive NIR FL/PA bimodal imaging nanoprobe (1‐NP) (Figure 3c).^[140]^ The probe consisted of an electrochromic material (1‐BrEt) as the ^•^OH‐reactive chromophore, which underwent significant color change during electron transfer (i.e., redox process). Initially, three electrochromic materials (1‐F‐Me, 1‐BrMe, and 1‐BrEt) were synthesized, revealing that 1‐BrEt exhibited the fastest reaction kinetics with ^•^OH. Subsequently, 1‐BrEt, NIR‐I fluorophore (NIR775), and NIR‐II fluorophore (IR1048) were encapsulated in micellar nanoparticles to form 1‐NP. Due to the absorption characteristics of 1‐Br Et below 500 nm, 1‐NP initially exhibited strong NIR‐I FL at 780 nm from NIR775 and NIR‐II FL at 1113 nm from IR1048, resulting in a relatively high FL_780_/FL_1113_ ratio. Meanwhile, 1‐NP displayed a weak PA signal at 755 nm, whereas IR1048 exhibited a strong PA signal at 905 nm, resulting in a relatively low PA signal between 755 and 905 nm (PA_755_/PA_905_). After reacting with ^•^OH, 1‐BrEt in 1‐NP oxidized to 2‐BrEt, which exhibited strong absorption at 767 nm. This oxidation quenched the NIR‐I FL of NIR775 due to the energy transfer from NIR775 to 2‐Br Et, while the NIR‐II FL of IR1048 at 1113 nm remained largely unchanged. Consequently, the FL_780_/FL_1113_ ratio of 1‐NP significantly decreased. Simultaneously, the PA signal of 1‐NP at 755 nm became activated, while the PA signal at 905 nm remained stable, resulting in an increased PA_755_/PA_905_ ratio. The FL/PA imaging capability of 1‐NP for detecting ^•^OH production was initially validated in Fenton reactions at the RAW264.7 cell level and in mice. Subsequently, 1‐NP was employed to monitor ^•^OH production during ferroptosis or tumor RT. Following intravenous injection, 1‐NP extravasated into tumor tissue due to the enhanced permeability and retention (EPR) effect. In pretreatment tumor cells with low ^•^OH levels, 1‐NP predominated in the tumor tissue, displaying a high FL_780_/FL_1113_ ratio but a low PA_755_/PA_905_ ratio (Figure 3d,e). Conversely, in the tumor cells treated with X‐ray radiation or erastin, elevated ^•^OH levels oxidized 1‐NP to 2‐NP, resulting in a low FL_780_/FL_1113_ ratio but a high PA_755_/PA_905_ ratio. These distinct changes in FL_780_/FL_1113_ and PA_755_/PA_905_ provided complementary signals, enabling 1‐NP to accurately detect ^•^OH levels during RT or ferroptosis, thereby minimizing false‐positive signals and facilitating early‐stage treatment monitoring. Additionally, IR1048 within 1‐NP facilitated in vivo delivery tracking with its ‘constant brightness’ NIR‐II FL at 1113 nm and PA signal at 905 nm, which proved beneficial for visualizing tumor tissue and guiding RT.

## 3 pH‐RESPONSIVE MOLECULAR PROBES

**FIGURE 4:**
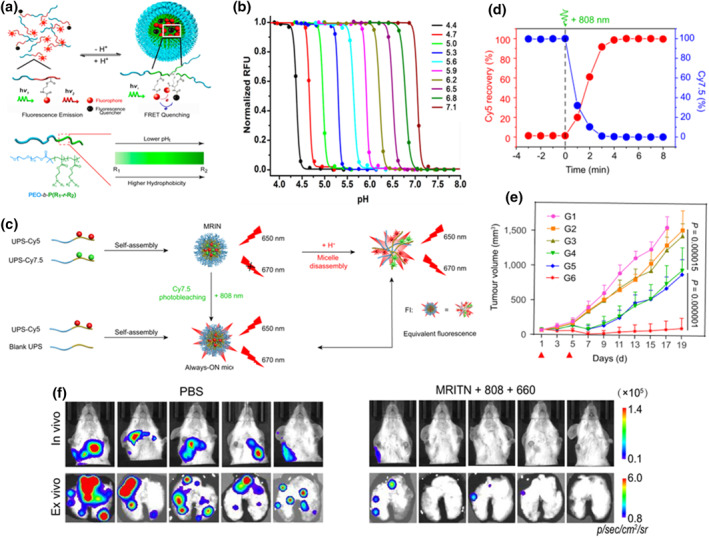
(a) Schematic design of UPS micellar nanoprobes and syntheses route. (b) Representative library of UPS nanoprobes with 0.3 pH increment covering the physiologic range of pH 4–7.4. Reproduced with permission. Reproduced with permission.^[143]^ Copyright 2014, American Chemical Society. (c) Schematic illustration of pH‐ and light‐triggered Cy5 FL recovery mechanisms. (d) Light‐triggered Cy5 fluorescence recovery and Cy7.5 photobleaching in MRIN. (e) Tumor growth curves of subcutaneous 4T1 tumor‐bearing mice with different treatments. (f) In vivo and ex vivo lung metastasis of each group analyzed by bioluminescence imaging (BLI). Reproduced with permission.^[144]^ Copyright 2022, The Author(s).

The tumor site is in acidic condition, which can be utilized for targeted drug delivery and specific imaging at the tumor site. However, conventional small molecule pH sensors, such as fluorescein and lysozyme sensors, were limited by a wide range of pH responses and weak FL emissions.[^141^, ^142^] Gao et al. reported the development of ultra‐pH‐sensitive (UPS) nanoprobes, which used block copolymers to design triamine‐containing monomers with precisely controlled hydrophobic substituents to tune different pH transitions.^[143]^ At low pH values, the micelles dissociated into cationic monomers with protonated ammonium groups (binding to PRA (ionizable tertiary amine block) fragments and emitting strong FL). As pH increased, the neutralized PR fragments became hydrophobic and self‐assembled into micelles, leading to FL quenching. Hydrophobic micellization significantly increased pH transitions (on/off state <0.25 pH units) (Figure 4a). Based on this design, they successfully distinguished the inter‐ventricular pH between early endosomes (6.0–6.5) and late endosomes/lysosomes (4.5–5.5) for siRNA delivery. They also used pH 6.9 nanoprobes for specific imaging in the acidic microenvironment of tumors. They further reported a strategy of fine‐tuning the hydrophobicity of PR fragments to control pH using two methyl methacrylate monomer copolymers with different hydrophobicities. In addition, FL quenching agents were introduced to expand the selection of dyes. Therefore, they established a UPS library that covered the entire physiological pH range of FL groups (400–820 nm) from 4 to 7.4 using predetermined pH conversion (Figure 4b). This study expands the application of small molecule pH sensors.

Based on previous work, Wang et al. developed a pH/light dual responsive monochromatic ratio imaging nanoparticle (MRIN) in 2022, which achieved quantitative distribution of nanomedicine inside and outside the cell.^[144]^ The nanoprobe was designed and synthesized using UPS nanotechnology, incorporating a blend of UPS‐Cy5 and UPS‐Cy7.5 FL polymers along with dye‐free polymers (Figure 4c). Due to the Förster resonance energy transfer (FRET) effect within the MRIN, the FL of Cy5 was quenched in the bloodstream and extracellular tumor environment (pH_e_∼6.7–7.1), transferring energy to Cy7.5. Upon internalization by tumor tissue cells, the acidic lysosomal environment (pHe∼6.0) caused nanoparticle dissociation, activating the Cy5 signal within MRIN. Conversely, the signal of extracellular NPs remained off, allowing quantification of nanoparticle internalization in tumor tissue. Subsequently, an external 808 nm laser was employed to irradiate the photodegradable FL quencher Cy7.5, thereby illuminating the Cy5 signal of extracellularly distributed NPs in tumor tissue (Figure 4d). This sequential illumination of intracellular and extracellular NP localization in the tumor area using non‐crosstalk stimuli enabled a more accurate quantification of their distribution. Additionally, they developed a monochromatic ratio imaging/therapeutic NP (MRITN) by blending UPS‐Ce6 polymer with an equal amount of UPS‐Cy7.5 polymer. Using this technology, they successfully analyzed the contribution of extracellularly distributed nanophotosensitizers to tumor treatment. The results demonstrated that compared to pure intracellular PDT, the combination of intracellular and extracellular PDT significantly inhibited tumor growth and metastasis by disrupting the tumor extracellular matrix and promoting tumor cell apoptosis, thereby maximizing the therapeutic efficacy of tumor treatment (Figure 4e,f). Based on these findings, the study speculated that the potential mechanism underlying the combined PDT anti‐metastasis effect involved the destruction of the extracellular matrix and downregulation of adhesion integrin β1. This research introduces a novel strategy for accurately quantifying the distribution of nanomedicines and designing multifunctional nanoplatforms.

**FIGURE 5:**
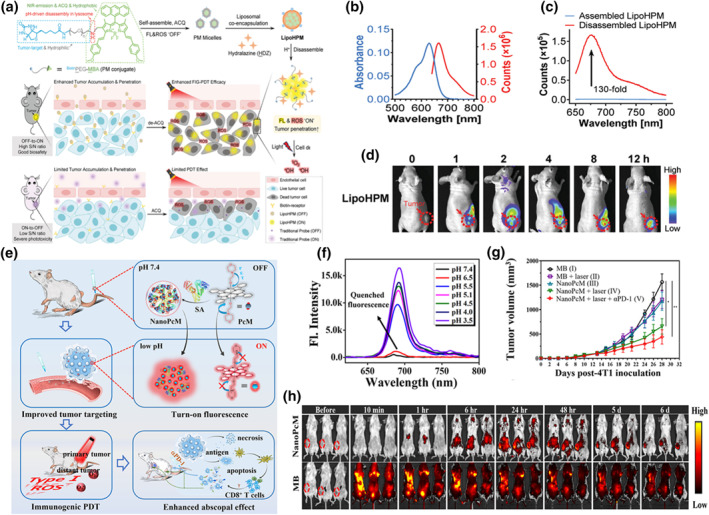
(a) Schematic illustration for the fabrication and in vivo delivery process of LipoHPM. (b) UV‐vis absorption and FL spectra of LipoHPM. (c) FL spectra of LipoHPM before and after dissociation. (d) In vivo FL imaging of nude mice bearing 4T1 breast tumors. Reproduced with permission.^[145]^ Copyright 2024, Wiley‐VCH GmbH. (e) Schematic diagram of the NanoPcM response process. (f) FL spectra of NanoPcM under different pH conditions. (g) Tumor growth curves of 4T1 tumor‐bearing BALB/c mice with different treatments. (h) In vivo FL imaging of NanoPcM in 4T1 tumor‐bearing mice. Reproduced with permission.^[146]^ Copyright 2022, American Chemical Society.

The employment of acidic pH of tumor lysosomes to activate FL and PDT therapy possesses great significance for precise treatment of tumors. The acid response strategy involves integrating protonated components into drug carriers to achieve pH response to acidic lysosomes. Concurrently, the cationic nature of drug carriers can induce the “proton sponge” effect, causing lysosomal swelling and eventual membrane rupture, thereby facilitating lysosomal escape and drug release. Moreover, lysosomal protonation of pH‐sensitive components (such as amino or dimethylamine groups) on photosensitizer promoted electron redistribution and charge separation, enhancing the type I and type II PDT. Recently, Liu et al. designed lysosomal acid‐responsive BDP NPs to activate FL and ROS specifically for FIG‐PDT applications.^[145]^ They introduced 4‐methyloxybenzaldehyde (contributing to NIR radiation) and anthracene (enhancing ROS generation) into the BDP scaffold, synthesizing the novel photosensitizer MeO‐BDPald (MBA). Subsequently, an acid‐sensitive amine linker connected the biotin‐modified hydrophilic polymer (BiotinPEG) with the hydrophobic photosensitizer (MBA), forming BiotinPEG‐MBA (PM) micelles via the ACQ effect. MBA was then covalently coupled with biotin‐modified polyethylene glycol (BiotinPEG), beneficial for tumor targeting and NP stability, through amino ligands, forming pH‐sensitive BiotinPEG‐MBA (PM) conjugates (Figure 5a). In aqueous media, the amphiphilic PM conjugates self‐assembled into fluorescent closed micelles, exhibiting the ACQ effect (Figure 5b,c). The hydrophobic MBA molecules aggregated tightly in the core, inhibiting FL and ROS production, while the hydrophilic BiotinPEG formed a stable shell, facilitating tumor targeting. The authors further designed and synthesized LipoHPM by co‐encapsulating HDZ and PM micelles within liposomes. Upon intravenous administration into 4T1 tumor‐bearing mice, the pre‐quenched PM micelles were effectively shielded by the liposomal shell, maintaining a low background signal and minimal phototoxicity in the bloodstream. Subsequently, LipoHPM actively targeted and accumulated in tumor cells overexpressing biotin receptors. The pH‐sensitive liposomes underwent decomposition in lysosomes, releasing encapsulated HDZ and PM micelles to perform their functions. Upon release, HDZ induced tumor cell arrest and reduced the solid tumor barrier, thereby enhancing NP penetration into tumors. Simultaneously, PM micelles underwent decomposition and escaped from lysosomes, achieving highly specific tumor imaging with a favorable signal‐to‐noise ratio and minimal side effects while maximizing PDT efficacy (Figure 5d). This study laid the groundwork for developing intelligently activated photosensitizers capable of high‐quality tumor imaging and potent PDT efficacy in future applications.

Additionally, Yoon and coworkers designed and synthesized a pH‐responsive PA nanomaterial (NanoPcM) for cancer treatment and imaging.^[146]^ NanoPcM was formed through strong interactions between phthalocyanine molecules grafted with morpholine and serum albumin (Figure 5e). The nitrogen atom in the morpholine structure possessed a lone‐pair electron. Due to the photoinduced electron transfer (PET) effect, the FL of the phthalocyanine silicon was quenched. However, under acidic conditions, the nitrogen atom readily protonated, disrupting the PET effect and restoring strong FL. It was verified that the FL quenching of NanoPcM resulted from both the PET effect at the molecular level and the ACQ effect at the supramolecular level. Therefore, under acidic conditions, the ACQ effect was eliminated by suppressing the PET effect and decomposing nanostructures, leading to strong FL at 691 nm (Figure 5f). Subsequently, the molecule's ability to generate ROS was investigated, demonstrating robust ROS production across different pH levels. It was also confirmed as a type‐I photosensitizer, indicating reduced impact of the hypoxic tumor microenvironment on NanoPcM's PDT properties. At the cellular level, it was speculated that NanoPcM entered cells via endocytosis, with the endosome serving as a carrier to transport it to lysosomes. The acidic environment of lysosomes (pH 4.5–5.0) likely protonated the nitrogen atom of the porphyrin, thereby restoring its FL. Finally, a 4T1 mouse model was employed to validate the imaging and therapeutic effects of NanoPcM in vivo (Figure 5g,h). NanoPcM effectively targeted tumors for FL imaging and combined PDT with αPD‐1 blockade synergistically to enhance therapeutic outcomes. In summary, utilizing the acidic pH microenvironment of tumor lysosomes can achieve specific fluorescence activation and PDT therapy, avoiding the occurrence of adverse side effects of drug therapy. Nevertheless, most photosensitizers face the problem of shallow tissue penetration depth of light, and there is an urgent need to develop excellent photosensitizers with longer‐response wavelength and deeper tissue penetration depth. Meanwhile, the study of lysosomal acidic pH is not yet precise enough, and further exploration is needed by researchers.

## 4 PHOTO‐RESPONSIVE MOLECULAR PROBES

**FIGURE 6:**
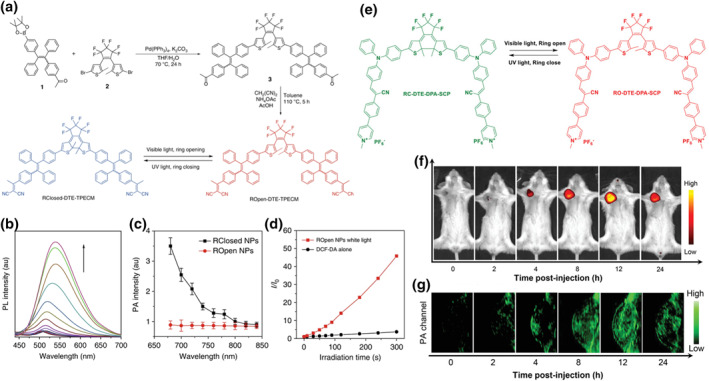
(a) Synthesis route of DTE‐TPECM molecule and its structural change under different light triggers. (b) FL spectra of RCclosed NPs after exposure to visible light (610 nm) for different times. (c) PA spectra of RClosed and ROpen NPs. (d) The relationship between *I*/*I*_0_ and irradiation time of ROpen NPs under white light irradiation. Reproduced with permission.^[156]^ Copyright 2018, The Author(s). (e) Reversible structure transformation of DTE‐DPA‐SCP molecule under different light irradiation. (f) FLI and (g) PAI after intravenous injection of RO NPs into 4T1 tumor‐bearing mice. Reproduced with permission.^[157]^ Copyright 2023, Wiley‐VCH GmbH.

Compared to stimuli like pH and temperature, light stimulation offers the advantage of spatiotemporal resolution and remote control, making it highly attractive for modulating molecules. Common photo‐responsive isomerization molecules include azobenzene,[^147^, ^148^, ^149^] and diarylethene.[^150^, ^151^] Azobenzene is an aromatic molecule with azo bonds at its core, exhibiting both cis‐metastable and trans‐stable structures. It can undergo reversible conversion upon heating or exposure to specific wavelengths of light, resulting in different photophysical properties.[^152^, ^153^, ^154^, ^155^] FLI boasts high sensitivity, while PAI offers good penetration depth. To achieve accurate diagnostic results, Qi et al. introduced a type of smart molecular probe with tunable photophysical properties.^[156]^ This molecule consisted of a photo‐controllable core of diarylethene (DTE) and a donor group of (1‐(4‐(1,2,2‐triphenylethylene)phenyl)ethyl)malononitrile (TPECM). DTE‐TPECM had both ring‐closed and ring‐open isomers. Under visible light irradiation, the DTE motif opened to form ROpen DTE‐TPECM, and under UV light irradiation, it reversibly closed to form RClosed DTE‐TPECM (Figure 6a). The geometric structure of ROpen DTE‐TPECM became more distorted in the ring‐open state, resulting in intermolecular interactions that significantly inhibited the non‐radiative decay pathway. Consequently, there was a strong emission peak at ∼550 nm for FLI (Figure 6b), and ROS could also be produced for PDT (Figure 6c). Due to the formation of a lower‐bandgap conjugated structure by RClosed DTE‐TPECM, the absorption peak red‐shifted to 650 nm. Additionally, RClosed DTE‐TPECM underwent intramolecular energy transfer, which greatly quenched FL in the ring‐closed form. The relatively planar geometry also enhanced the intermolecular interactions, boosting the PAI properties (Figure 6d). Experimental verification showed that the molecule could reversibly switch between ring‐closed and ring‐open forms and exhibited good stability. To increase the targeting ability, a peptide (YSA) capable of tightly binding to the highly expressed EphA2 protein in cancer cells was modified on the nanoprobe surface. The nanoprobe performed well in PAI before surgery, and converted to the ROpen DTE‐TPECM form for FL imaging and PDT during surgery. This binding mode significantly reduced the risk of in situ tumor recurrence. In cases of debulking surgery where complete tumor resection was not achieved, PDT with ROpen YSA NPs successfully extended the lifespan of mice.

Smart photosensitizers that could optimize photophysical energy conversion and related phototherapy performance through optically driven structural switches have promising application prospects in the multifunctional biomedical field. Ding and co‐workers designed and synthesized a light‐controlled “all‐in‐one” molecule for multimodal imaging‐guided surgery and tumor immunotherapy.^[157]^ The molecule consisted of two parts with the core DTE structure used for light control. The two end groups were DPA‐SCP, endowing the molecule with AIE characteristics and high ROS generation efficiency (Figure 6e). This molecule had a strong PA signal in the ring‐closed RC‐DDS for preoperative PAI, while the ring‐open RO‐DDS exhibited strong FL emission (∼630 nm) and ROS generation under visible light irradiation for intraoperative surgical navigation and PDT. It was validated in tumor‐bearing mice, demonstrating that the combination of PAI and FLI could significantly improve tumor surgery outcomes(Figure 6f,g). The ROS generated by photosensitizers not only inhibited tumor growth but also induced strong immunogenic cell death (ICD). Therefore, further research was conducted on the anti‐tumor effects and potential immune system stimulation of RO‐DDS molecules. They established a 4T1 tumor‐bearing model and simulated a residual tumor model post‐surgery. By measuring immune‐related factors in vivo, it was verified that this molecule effectively stimulated the immune system and inhibited the growth of metastatic tumors, showing great long‐term anti‐tumor potential. The above work designed and synthesized a new type of photo‐controlled molecule, providing novel approaches for precise multimodal imaging and treatment.

## 5 HYPOXIA‐RESPONSIVE MOLECULAR PROBES

**FIGURE 7:**
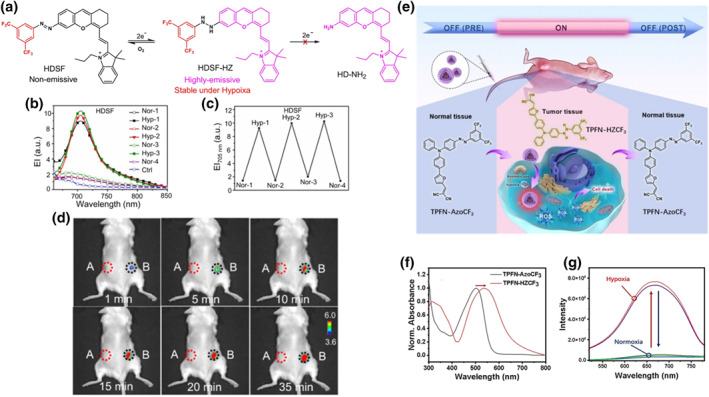
(a) The reversible hypoxia response mechanism of molecular probe HDSF. (b) FL spectra and (c) corresponding FL intensity of HDSF at 705 nm upon hypoxia and normoxia treatment. (d) HDSF was injected into human breast MCF‐7 tumor xenograft mice for different durations of bright field and FLI. Reproduced with permission.^[164]^ Copyright 2021, The Author(s). (e) The reaction mechanism of TPFN‐AzoCF_3_ under normoxic and hypoxic conditions. (f) Absorption and (g) FL spectra of TPFN‐AzoCF_3_ under normoxic and hypoxic conditions. Reproduced with permission.^[165]^ Copyright 2023, Wiley‐VCH GmbH.

Hypoxia is considered as the typical microenvironment characteristic within multiple solid tumors, posing significant challenges to various treatments.[^158^, ^159^, ^160^, ^161^] Sensitive hypoxia imaging not only helps clinicians accurately diagnose lesions but also aids in selecting the most effective treatment strategy, thereby allowing for monitoring and improving treatment outcomes. Some methods had been reported for hypoxia detection, such as nitroreductase and hypoxia‐inducible factors. However, these methods faced the drawback of being “always on” or “always off” and lacked reversibility, making them unsuitable for in vivo hypoxia detection.[^162^, ^163^] To achieve specific FLI in hypoxic regions, He et al. reported an aromatic azo FL probe that could reversibly respond to hypoxia.^[164]^ The probe HDSF consisted of two parts: one part was a xanthene cyanine fused fluorophore (HD), which targeted mitochondria and was used for NIR FLI, and the other part was an oxidation‐reduction inert electron‐withdrawing group, 3,5‐difluoromethylbenzene (Figure 7a). These two parts were connected by an azo bond that responded to hypoxia. Under normal oxygen conditions, the azo aryl groups exhibited almost no FL due to rapid photo‐induced *E*‐*Z* isomerization. By modifying the aryl azo group with an electron‐withdrawing group (‐CF_3_), the intermediate could be stabilized, increasing the activation energy required for subsequent reduction. This modification prevented the irreversible reduction of hydrazine, enabling reversible reactions under hypoxia and achieving FL activation under anaerobic conditions. The reversible reduction mechanism of HDSF was explored through density functional theory (DFT) calculations. By calculating the energy of intermediates and products during the reduction process of HDSF, it was further verified that using electron‐withdrawing groups to replace aromatic azo to construct FL groups was an effective strategy for designing reversible hypoxia probes. Under hypoxic conditions, the absorption peak of HDSF shifted from 650 to 700 nm with a clear emission peak at 705 nm (Figure 7b). Additionally, it underwent multiple stable reversible transitions under normoxic and hypoxic conditions (Figure 7c). HDSF imaging in mice further confirmed that the molecular probe performed well in FLI under hypoxic conditions (Figure 7d).

Liu et al. designed and synthesized a convertible photosensitizer molecule, TPFN‐AzoCF_3_, with an “OFF‐ON‐OFF” function, providing comprehensive protection before and after PDT and activating only in a tumor hypoxic environment, thus greatly reducing the toxic side effects of PDT.^[165]^ TPFN‐AzoCF_3_ consisted of two parts: a type I photosensitizer with a D‐A structure (TPFN) and a heteroxia‐normoxia cyclic response group (AzoCF_3_). The aromatic azo group underwent reversible structural transformation under normoxic and hypoxic conditions. Under normoxic conditions, the rapid *E*‐*Z* isomerization under light irradiation effectively consumed the excited state energy of the photosensitizer, preventing it from producing ROS and FL (Figure 7e). It could be activated only in hypoxic tumors, where the aromatic azo group was converted to a hydrazine group (TPFN‐HZCF_3_), leading to FL activation and ROS production. The absorption maximum of TPFN‐AzoCF_3_ after hypoxia treatment shifted from 500 to 550 nm (Figure 7f), and FL emission appeared at 671 nm. Furthermore, the ability of this molecule to generate ROS before and after hypoxia response was investigated. Under normal oxygen conditions, minimal ROS production was observed, but a significant amount of ROS was generated following hypoxia‐induced structural changes (Figure 7g). It was confirmed that the ROS produced was predominantly O_2_^−^, indicating that it was type‐I photosensitizer and the hypoxic tumor microenvironment minimally affected PDT efficacy. Hypoxia imaging and hypoxia reoxygenation synthesis imaging were conducted in mice, revealing strong FL signals exclusively in hypoxic tumor regions. In the HeLa xenograft mouse model, the anti‐tumor efficacy was assessed, demonstrating that the experimental group effectively suppressed tumor growth, thereby validating the molecule's potent PDT therapeutic effect.

**FIGURE 8:**
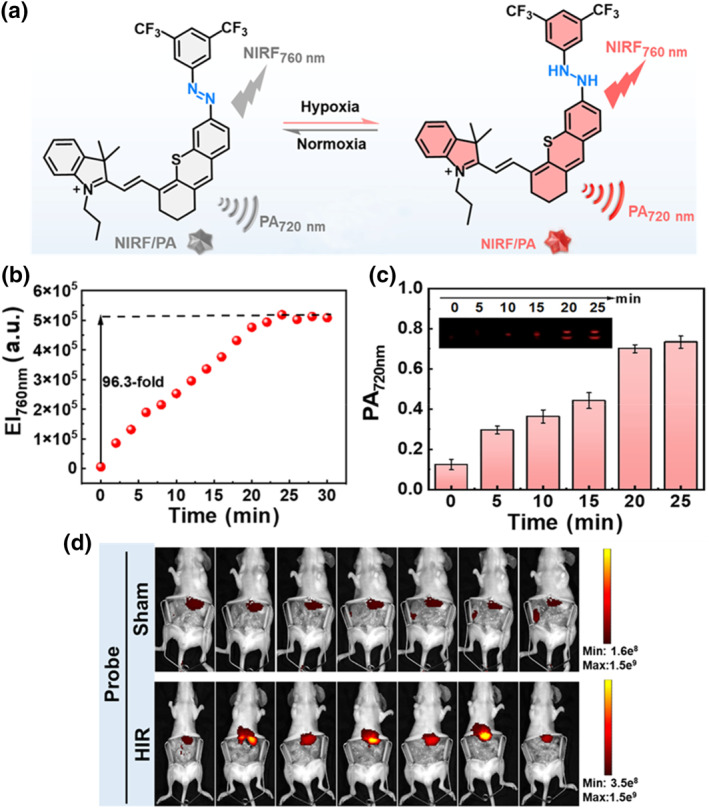
(a) The reaction mechanism of AzoCys‐CF_3_ under normoxic and hypoxic conditions. The changes of (b) FL intensity at 760 nm and (c) PA signal of AzoCys‐CF_3_ at 720 nm over time under hypoxic conditions. (d) FLI of the liver ischemia‐reperfusion mice at 2 h after injection of AzoCys‐CF_3_. Reproduced with permission.^[166]^ Copyright 2024, Chinese Chemical Society.

Recently, Chen et al. reported a reversible hypoxia probe (AzoCys‐CF_3_) for FL and PA dual‐mode imaging of acute and chronic hypoxic liver injury (Figure 8a).^[166]^ AzoCys‐CF_3_ consisted of two parts, with thioanthracene semi‐anthocyanin as the NIR FL group. Due to the electron‐withdrawing properties of trifluoromethyl, the aromatic azo‐coupled trifluoromethyl quenched the FL, acting as a hypoxia‐responsive group and allowing the molecule to undergo reversible bioreduction/oxidation in the hypoxia‐reoxygenation cycle. Under normoxic conditions, the maximal absorption of AzoCys‐CF_3_ was at 625 nm, while under hypoxic conditions, there was a significant red shift with the absorption peak around 750 nm. Due to the FL quenching effect, the probe only had weak FL under normoxic conditions. However, under hypoxic conditions, a strong emission peak appeared at 760 nm, and the probe's FL periodically lit up and extinguished with the oxygen content, exhibiting good stability within a certain period (Figure 8b). The PA signal of AzoCys‐CF_3_ at 720 nm also increased with the decrease of oxygen content, achieving a reversible dual‐mode imaging probe (Figure 8c). Previous literature reported that anthocyanin dyes tended to accumulate in the liver, ensuring the feasibility of their applications in acute and chronic hypoxic liver injury. Next, the ability of the AzoCys‐CF_3_ molecule to image cellular hypoxia in liver L02 normal cells and HepG2 cancer cells was verified. The experimental results at the molecular level showed that the FL of cells was very weak under normoxic conditions, but the FL intensity significantly increased as the oxygen content decreased. When the normoxic environment was restored, the FL inside the cells gradually disappeared. A model of HepG2 cells subcutaneously implanted in BALB/c mice was constructed, and AzoCys‐CF_3_ was injected into the tumor and the subcutaneous tissue on the other side. The FL intensity inside the tumor was significantly stronger than that under the skin, and the PA intensity in the tumor area was about 4.0 times higher than that on the subcutaneous side of the abdomen. Subsequently, chronic liver hypoxia mouse models were constructed for liver ischemia‐reperfusion and non‐alcoholic fatty liver disease. Significant signals were detected in the hypoxic area using the in vivo imaging system and PAI. These results indicated that AzoCys‐CF_3_ was capable of monitoring acute liver hypoxia in real‐time for HIR and NIRF (Figure 8d). The proposal of the smart “OFF‐ON‐OFF” strategy offers a new design approach for creating photosensitizers with fewer side effects that can be activated on demand in the future.

## 6 ENZYME‐RESPONSIVE MOLECULAR PROBES

**FIGURE 9:**
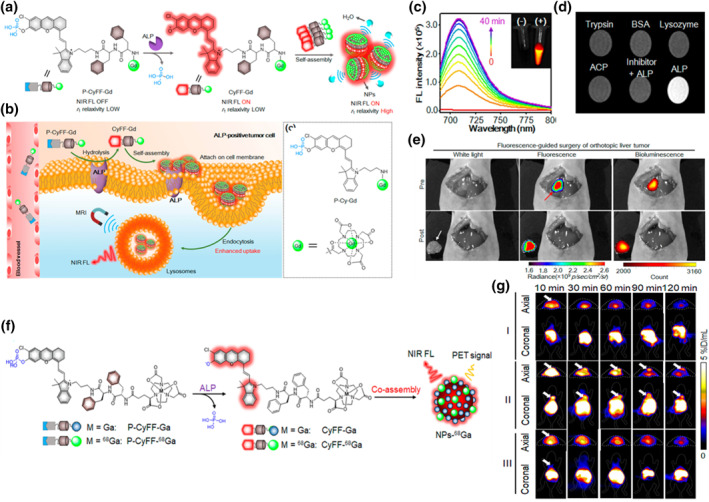
(a) Chemical structure of P‐CyFF‐Gd and proposed ALP‐mediated fluorogenic reaction and in situ self‐assembly of P‐CyFF‐Gd into NPs that show increased NIR FL and r_1_ relaxivity. (b) The proposed mechanism of P‐CyFF‐Gd for NIR FL/MR bimodality imaging of ALP‐positive tumor cells in vivo and chemical structure of the designed non‐assembled control probe P‐Cy‐Gd. (c) FL spectra of P‐CyFF‐Gd incubated with ALP. (d) T_1_‐weighted MRI of P‐CyFF‐Gd incubated with trypsin, BSA, lysozyme, ACP, ALP or ALP together with its inhibitor Na_3_VO_4_ in Tris buffer. (e) Bimodality imaging and FL‐guided surgery of orthotopic liver tumors. Reproduced with permission.^[169]^ Copyright 2019, American Chemical Society. (f) The structures of P‐CyFF‐^68^Ga and P‐CyFF‐Ga and the proposed formation of fluorescent and radioactive NPs‐^68^Ga via ALP‐triggered in situ co‐assembly. (g) FLI of HeLa tumor in mice. Reproduced with permission.^[170]^ Copyright 2021, American Chemical Society.

Multimodal molecular imaging probes play a crucial role in biomedical and clinical research. However, many probes exhibit “always‐on” signals, which can result in a poor tumor background ratio and may not meet the requirements for early diagnosis of malignant tumors. Tumor usually expresses highly specific enzymes that play a crucial role as the important molecular targets in tumor development and treatment. Developing tumor enzyme responsive molecular probes with high sensitivity and specificity has become one of the current research hotspots.[^167^, ^168^] Ye et al. designed and synthesized an NIR FL/MRI dual‐mode small molecular probe that could be activated for in vivo imaging through an enzyme‐mediated FL reaction and in situ self‐assembly.^[169]^ The probe comprised three components: a NIR cyanine fluorescent group with a phosphate group recognized by alkaline phosphatase (ALP), a paramagnetic DOTA‐Gd chelate for MRI, and a hydrophobic dipeptide Phe‐Phe (FF) linker to facilitate self‐assembly (Figure 9a). Initially, P‐CyFF Gd was a water‐soluble small molecular probe due to the presence of hydrophilic PO_3_H and DOTA‐Gd groups, displaying quenched NIR FL and a low *r*_1_ relaxation rate. Upon systemic administration, its small size and hydrophilic nature facilitated its distribution into tumor tissues (Figure 9b). In ALP‐positive tumor tissues, dephosphorylation of P‐CyFF Gd resulted in CyFF Gd activation, leading to 710 nm NIR emission. Simultaneously, the FF dipeptide within CyFF Gd could promote the intermolecular interactions and self‐assembly into NIR fluorescent and magnetic NPs. Self‐assembling NPs easily anchored to cell membranes, promoting endocytosis and transport to lysosomes in tumor cells, thereby increasing their retention in ALP‐positive tumor cells, while inactive P‐CyFF Gd might be cleared. Subsequently, they demonstrated that P‐CyFF Gd could be specifically activated by ALP in vitro (Figure 9c), resulting in nearly a 70‐fold increase in FL intensity at 710 nm. Upon self‐assembly of P‐CyFF Gd into NPs, the T_1_ relaxation time of water molecules gradually shortened, producing brighter T_1_‐weighted MRI (Figure 8d). Further investigations revealed that P‐CyFF Gd could effectively undergo dephosphorylation by membrane‐bound ALP in HeLa cells and cell microspheres, forming NPs that emitted strong NIR FL and MR signals. Finally, they utilized P‐CyFF Gd to visually guide the resection surgery of HepG2 liver tumors in situ. By utilizing the stronger NIR and MR signals in tumor tissue compared to normal tissue, they accurately delineated the tumor edges in the liver, demonstrating that P‐CyFF Gd was a promising molecular imaging tool for guiding the resection of HepG2 liver tumors (Figure 8e). Thus, the strategy combining enzyme‐mediated FL reaction with in situ self‐assembly provided a framework for designing activatable probes based on small molecule activatable NIR FL/MRI bimodal probes and other synergistic imaging modalities. Subsequently, Ye's research team applied this strategy to design and synthesize CyFF‐^68^Ga and CyFF Ga, which could be dephosphorylated under the action of ALP, and then co‐assembled them into fluorescent and radioactive nanoparticles (NP‐^68^Ga) (Figure 9f).^[170]^ The NP‐68Ga formed in situ upon ALP triggering was readily anchored on the membrane of ALP‐positive HeLa cells, enabling the simultaneous enrichment of NIR FL and radioactivity, thereby achieving NIR light/PET dual‐mode imaging (Figure 9g). In summary, ALP enzyme‐mediated bimodal probes can effectively guide the resection of tumor tissue. The strategy of combining enzyme‐mediated fluorescence reactions with in situ self‐assembly for drug delivery and time‐controlled release in tumors is crucial for improving patients' treatment outcomes.

**FIGURE 10:**
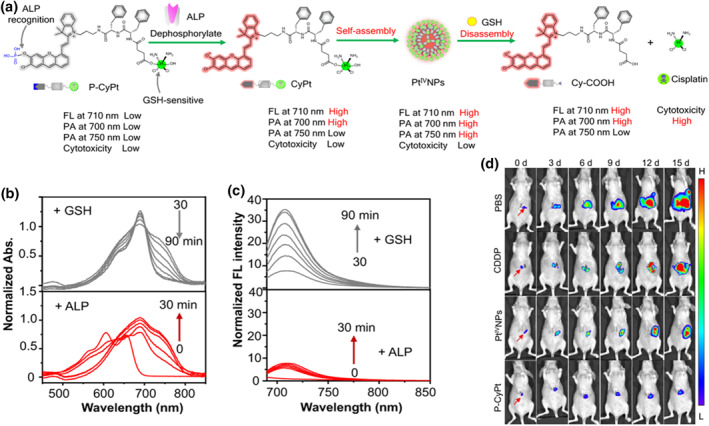
(a) Chemical structure of P‐CyPt and the ALP‐triggered dephosphorylation and in situ self‐assembly into PtIVNPs, followed by GSH‐triggered disassembly to release cisplatin and Cy‐COOH. (b) Absorption and (c) FL spectra ofP‐CyPt with the treatment of GSH and ALP for different times. (d) Representative BLI and FLI of liver tumor following indicated treatments. Reproduced with permission.^[172]^ Copyright 2023, The Author(s).

Based on prior research, Ye et al. documented a sequential stimulus‐triggered in situ self‐assembly and disassembly approach aimed at guiding therapeutic drug delivery and release in vivo.^[171]^ They engineered a stimulus‐responsive small molecule cisplatin prodrug, P‐CyPt, incorporating a NIR anthocyanin fluorescent group (mCy) with an ALP‐recognized phosphate group (‐PO_3_H), a GSH‐reducible CDDP prodrug (Pt(IV)), and a hydrophobic D‐Phe D‐Phe (FF) dipeptide. Initially, the probe exhibited weak NIR FL, a low PA signal, and minimal cytotoxicity (Figure 10a). Following systemic administration, P‐CyPt, as a small molecule, could extravasate and permeate into tumor tissues. Within membrane‐bound ALP‐positive tumor tissues, ALP promptly dephosphorylated P‐CyPt to generate CyPt, thereby activating NIR FL (λ_em_ = 710 nm) and PA signal (*λ* = 700 nm) (Figure 10b,c). More significantly, CyPt, with enhanced hydrophobicity, readily self‐assembled into Pt (IV) NPs (Pt^IV^NPs) due to aggregation, resulting in a red shift that activated an additional PA signal (*λ* = 750 nm). The authors initially explored the in situ self‐assembly behavior of P‐CyPt triggered by ALP and the in vitro disassembly behavior driven by GSH. It was observed that P‐CyPt could form NPs with an average diameter of 160 nm upon ALP activation, exhibiting significant FL alterations and PA activation. The authors investigated the morphology of Pt^IV^NPs and observed changes in signal intensity at 710 nm for FL and 750 nm for PA, confirming that GSH effectively dissociated Pt^IV^NPs and triggered the release of Cy‐COOH and Pt (II) drugs. Cytotoxicity analysis demonstrated that P‐CyPt exhibited superior permeability compared to pre‐fabricated Pt^IV^NPs, thereby more effectively targeting deep tumor cells. Next, the authors examined P‐CyPt's ability to guide the treatment of s.c. tumors using FL and PA dual‐mode imaging. It was observed that P‐CyPt primarily formed Pt^IV^NPs in HeLa tumor tissue at 2 h and largely decomposed within the tumor by 4 h. P‐CyPt‐treated tumors exhibited the highest injected dose per gram of tissue (%ID/g) among all excised organs, while Pt^IV^NPs and CDDP led to significant side effects; P‐CyPt effectively mitigated these effects in mice and demonstrated potent therapeutic efficacy against s.c. HeLa tumors. The author further applied P‐CyPt for imaging‐guided treatment of HepG2/Luc liver tumors in situ and found that P‐CyPt could be effectively activated to produce strong NIR FL, accurately locating liver tumors in situ, which was more effective than pre‐formed Pt^IV^NPs. Under the guidance of FLI, the location of liver tumors could be clearly delineated (Figure 10d). Therefore, in situ self‐assembly triggered by ALP and intracellular disassembly of P‐CyPt triggered by GSH could temporally control the transmission and release of cisplatin in tumors, significantly improving anti‐tumor efficacy and reducing off‐target toxicity in HepG2 liver tumor mice in situ. P‐CyPt also demonstrated high feasibility in the joint detection of tumor lesions and non‐invasive monitoring of drug release through NIR FLI and dual PAI. This strategy provides insights for developing other stimulus‐responsive small molecule therapeutic probes to enhance imaging and treatment of cancer and other malignant diseases. The use of stimulus‐responsive small molecule probes to evaluate cancer response to therapy holds significant promise for improving treatment efficacy and optimizing treatment timing in cancer patients. Although P‐CyPt is triggered by the ALP enzyme and GSH to deliver and release cisplatin, it reduces off target toxicity of the drug, avoids systemic toxicity, and significantly improves anti‐tumor efficacy. However, prolonged use of cisplatin can lead to the development of drug resistance, and the design of our small molecule therapy probes should pay attention to the issue of drug resistance in the future. At the same time, inducing ICD of tumor cells and overcoming the immunosuppressive environment of tumor tissue can more effectively kill primary tumor cells, inhibit tumor recurrence and metastasis, and improve therapeutic efficacy. However, how to construct tumor‐specific sensitizers to generate sonodynamic, photodynamic, and immune combination therapy effects still faces challenges.

**FIGURE 11:**
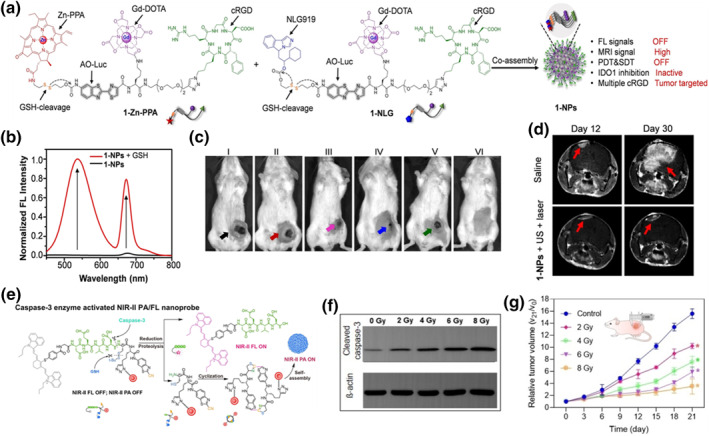
(a) Chemical structures of 1‐Zn‐PPA and 1‐NLG. (b) FL spectra of 1‐NPs incubated with or without GSH. (c) Representative photographs of mice after various treatments. (d) Representative T_1_‐weighted MRI of GL261/Luc gliomas. Reproduced with permission.^[172]^ Copyright 2023, Wiley‐VCH GmbH. (e) Caspase‐3 enzyme activated NIR‐II PA/FL nanoprobe. (f) Western blot analyses of the cleaved caspase‐3 for cell apoptosis in HepG2 tumor cells after various treatments. (g) The relative tumor growth curves with different treatments. Reproduced with permission.^[174]^ Copyright 2021, Wiley‐VCH GmbH.

In order to achieve multimodal imaging and collaborative acousto‐optic dynamic immunotherapy, Ye et al. reported a tumor‐targeted and redox‐activated nanosensitizer (1‐NPs), which was used for acousto‐optic dynamic immunotherapy of tumors through molecular co‐assembly and redox‐controlled disassembly.^[172]^ This approach addressed the challenge of low ROS production efficiency and the difficulty in completely inhibiting tumor growth with single acousto‐optic therapy. 1‐NPs were synthesized by simply co‐assembling two amphiphilic small molecule probes (1‐Zn‐PPA and 1‐NLG) with an optimized molar ratio (1/1.1) of 1‐Zn‐PPA to 1‐NLG. Both molecules shared the same molecular components, including the hydrophobic aminoluciferin (AO Luc) fluorophore, the hydrophilic Gd‐DOTA chelating MRI agent, the tumor‐targeting ligand cRGD, and GSH‐sensitive disulfide bonds. Additionally, 1‐Zn‐PPA contained NIR zinc‐chelated phospholipids (Zn‐PPA), while 1‐NLG included IDO1 inhibitors (NLG919) (Figure 11a). In an aqueous solution, 1‐Zn‐PPA and 1‐NLG co‐assembled into spherical NPs, exhibiting quenched dual FL emission at 547 and 672 nm, respectively, along with inhibited sonodynamic therapy (SDT) and PDT activities coupled with a high r1 relaxation rate. Moreover, the NLG919 molecules within 1‐NPs were shielded and unable to inhibit IDO1. After systemic administration to tumor‐bearing mice, 1‐NPs targeted tumors via cRGD binding, allowing non‐invasive tracking through T_1_‐weighted MRI. Within tumor cells, the highly expressed GSH reduced disulfide bonds in 1‐NPs, leading to their breakdown and the release of small molecules including 2‐Gd, ZnPPA‐SH, and NLG919. Released Zn‐PPA‐SH bound to endogenous albumin, activating AO Luc FL at 547 nm and ZnPPA NIR FL at 672 nm (Figure 11b), thereby initiating SDT and PDT activities. Simultaneously, the released NLG919 inhibited IDO1 activity. Guided by enhanced MRI and dual FL signals, ultrasound and a 671 nm NIR laser irradiated 4T1 tumor tissue in mice, generating cytotoxic ROS to eliminate tumor cells and induce ICD to enhance tumor immunogenicity (Figure 11c). Furthermore, the authors evaluated acoustic‐PIT using the orthotopic GL261/Luc glioma mouse model, demonstrating that 1‐NPs also exerted potent effects via cRGD‐mediated tumor‐targeted delivery and GSH‐triggered decomposition (Figure 10d). The molecular co‐assembly and controllable disassembly strategies designed in this study are expected to serve as a universal platform for constructing tumor cell targeted and activated organic nano sensitizers, which can be applied to multimodal imaging and multimodal combination therapy of other tumors. Small molecule fluorescent probes are also commonly used to monitor the therapeutic effect of tumors, such as the use of stimulus responsive small molecule probes to evaluate the response of cancer to RT is of great value in improving the treatment efficacy of cancer patients and optimizing treatment time.^[173]^

Song et al. developed a nanoprobe, AuNP@DEVD‐IR1048, designed to be activated by caspase‐3 for early assessment of RT efficacy.^[174]^ This nanoprobe consisted of NIR‐II FL molecule IR‐1048 as the energy donor and NIR‐I core‐shell structured gold NPs (AuNPs) as PA contrast agents. The connection between the energy donor and acceptor via caspase‐3 specific peptide substrate (DEVD) achieved high FL quenching efficiency through the FRET effect. Consequently, the probe exhibited weak FL signal and negligible PA signal in the NIR‐II region. Upon exposure to caspase‐3 enzyme, the amide bond linking DEVD and Cys on AuNP@DEVD‐IR1048 was cleaved, releasing IR‐1048 and activating 1150 nm NIR‐II FL emission. This process also produced Cys (StBu)‐(AuNP)‐CBT. Subsequent reduction of the disulfide bond by GSH triggered a CBT Cys click condensation reaction, forming cyclized dimers that self‐assembled into aggregates of AuNPs (Figure 11e). This aggregation activated a 1250 nm NIR‐II PA signal due to plasma coupling effects between adjacent AuNPs. After uptake by HepG2 cells, the nanoprobes induced a significant increase in ROS production due to RT, activating caspase‐3 and resulting in NIR‐II FL and PA signals. This process also caused disruption of DNA double strands, leading to apoptosis of cancer cells. The researchers observed that activated caspase‐3 levels rose with increasing X‐ray irradiation dose, correlating positively with enhanced NIR‐II PA or FL intensity. They then explored using cleaved caspase‐3 as a bridge between imaging signals and treatment efficacy, establishing a new evaluation framework based on NIR‐II FLI/PAI for self‐assessment and early prediction of RT efficacy (Figure 11f). They examined the relationship between caspase‐3 expression levels and tumor shrinkage rates in irradiated mice across different doses, discovering a negative correlation between imaging signals and tumor volume (Figure 11g). Finally, they validated the application of AuNP@DEVD‐IR1048 in situ liver cancer models, confirming a close correlation between changes in liver tumor volume and ΔPA or ΔFL intensity. Thus, this novel live imaging strategy holds promise for monitoring caspase‐3 expression activated by RT, suggesting avenues for developing stimulus‐responsive probes for early evaluation of therapeutic effects using other biomarkers.

**FIGURE 12:**
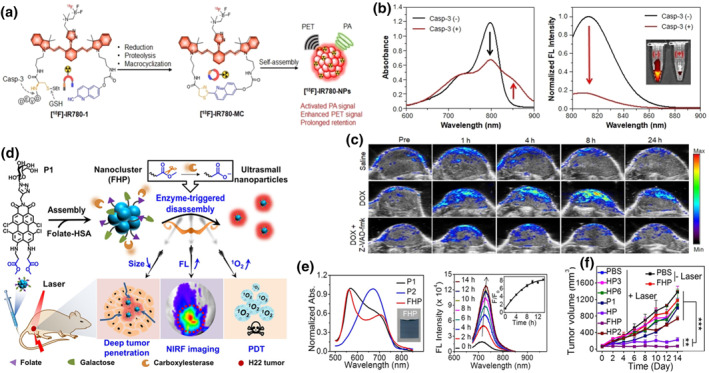
(a) Chemical structure of the designed PAI/PET bimodal probe [^18^F] IR780‐1 and its macrocyclization and self‐assembly targeting Casp‐3 and GSH. (b) Absorption and FL spectra of IR780‐1 before and after incubation with Casp‐3. (c) PAI of tumor‐bearing mice with different treatments. Reproduced with permission.^[175]^ Copyright 2022, Wiley‐VCH GmbH. (d) Schematic diagram of FHP nanocluster manufacturing. (e) Normalized absorption spectra of P1, P2, and FHP, and FL spectra when incubated with CE for different times. (f) Tumor volumes of H22 tumor‐bearing mice with different treatments. Reproduced with permission.^[176]^ Copyright 2020, Wiley‐VCH Verlag GmbH & Co. KGaA, Weinheim.

Positron emission tomography (PET) provides whole‐body imaging with high sensitivity and deep tissue penetration but low resolution. PAI, on the other hand, offers high resolution. Ye et al. developed a PA probe based on the CHQ‐Cys macrocyclization reaction in cells to achieve tumor targeting and Casp‐3 activation.^[175]^ To achieve dual‐activated imaging combining PET and PA, they made three modifications to the basic framework of triazole‐IR780 and [^18^F]‐IR780‐1. Initially, triazole‐IR780 was utilized for PAI. Subsequently, they incorporated 2‐cyano‐6‐hydroxyquinoline (CHQ), a D‐Cys peptide substrate (DEVD) terminated with a Casp‐3 cleavable material, a GSH‐reducible disulfide, and 18F‐labeled zwitterionic organotrifluororate ([^18^F]‐AMBF3) for PET imaging (Figure 12a). Upon delivery of [^18^F]‐IR780‐1 to the tumor site, DEVD protein hydrolysis mediated by Casp‐3 generated an amino group, GSH induced reduction of disulfide bonds, and CHQ underwent spontaneous intramolecular cyclization, forming a large ring [^18^F]‐IR780‐MC. The rigid structure and strong hydrophobicity of the large ring facilitated strong intermolecular interactions, promoting self‐assembly into NPs ([^18^F]‐IR780‐NPs) that reduced diffusion and prolonged residence time near the tumor. After the formation of [^18^F]‐IR780 NPs, the aggregation of IR780 within the NPs led to the ACQ effect, releasing energy through radiative transitions and significantly enhancing the signal of PA. Additionally, the accumulation of enriched ^18^F enhanced the PET effect of IR780 NPs. We successfully utilized the self‐assembly of NPs to enhance PA and PET signals after Casp‐3 response at the tumor site, enabling more accurate and comprehensive imaging analysis. Comparing optical signals with and without Casp‐3 at the molecular level revealed that Casp‐3 addition caused a decrease in the absorption peak at 795 nm and a new peak at 855 nm appeared. The emission peak at 813 nm decreased significantly, while the PA signal at 790 nm was greatly enhanced (Figure 12b). This confirmed that Casp‐3 addition induced the ACQ effect of IR780 after self‐assembly. Therefore, this molecule could be used for dual‐mode imaging of Casp‐3 activity and distribution in DOX‐induced apoptotic tumors in live mice. Based on this research, a DOX‐induced tumor apoptosis model was established in U87MG tumor‐bearing mice, and dual‐mode imaging was performed using the [^18^F]‐IR780‐1 molecule. Compared to the control PBS group, the experimental group exhibited significant PET and PA signals, with the PA signal peaking at 8 h post‐injection, showing a 21.7‐fold increase (Figure 12c). Thus, [^18^F]‐IR780‐1 serves as a sensitive probe for dual‐mode imaging of PET and PA in tumor apoptosis.

Yin et al. reported a carboxylesterase (CE)‐responsive nanocluster with switchable particle size, enhanced FL, and improved PDT.^[176]^ Perylene monoimide (PMI) dyes are frequently used in light‐based self‐assembly systems due to their extensive *π*‐conjugated systems. Building on this, they designed and synthesized a CE‐responsive PMI nanocluster (P1), where tetrachloroperylene monoimine served as the core of the system, surrounded by *β*‐alanine methyl as the substrate and electron‐donating group for CE. Galactose was introduced at the imine position to target tumors and enhance hydrophilicity. Finally, P1 was assembled with folate‐modified albumin to form a nanostructure (FHP), which exhibited improved biocompatibility and enhanced accumulation at the tumor site (Figure 12d). TEM characterization revealed that the particle size of FHP was approximately 100 nm, which reduced to approximately 10 nm upon CE‐responsive disassembly. The large particle size of FHP prolonged its retention time in the bloodstream, and upon disassembly, it facilitated deeper penetration of NPs into tumors, thereby enhancing the therapeutic effect of PDT. Subsequently, the optical properties and PDT effect of the nanoclusters were investigated. It was found that after the addition of CE, the FL intensity of FHP increased approximately eightfold, and its ability to generate ^1^O_2_ increased by about fourfold (Figure 12e). This indicated that CE could disassemble FHP into ultra‐small nanoparticles, enhancing both its FL intensity and PDT effects. Further validation through cell and live experiments demonstrated that at the cellular level, FHP extended from the periphery to the center of tumor spheres, whereas in the control group, it remained primarily on the surface. They established an H22 tumor‐bearing mouse skin model to observe the in vivo phototherapy effects of FHP. FLI results confirmed significant targeting capability and enhanced aggregation of FHP at the tumor site. Phototherapy effectively suppressed tumor growth in the experimental group (Figure 12f). These results affirmed that FHP exhibited effective NIRF imaging guidance, activatable properties, and deep PDT effects, which are highly significant for guiding the development of new photosensitizers and stimulus‐responsive nanomedicines for future cancer treatments.

## 7 H_2_S‐RESPONSIVE MOLECULAR PROBES

**FIGURE 13:**
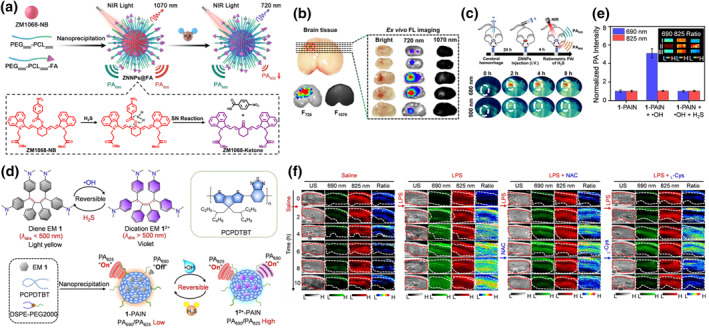
(a) The composition of ZNNPs@FA and H_2_S detection mechanism. (b) FLI at 720 and 1070 nm of brain tissues injected with ZNNPs. (c) PAI of the brain region treated with ZNNPs. Reproduced with permission.^[182]^ Copyright 2022, The Author(s). (d) Chemical structures of EM1 and PCPDTBT and the reversible response mechanism of EM1 under redox conditions. (e) PA intensity of 1‐PAIN under different treatments. (f) After different treatments on mouse liver, images of 1‐PAIN in PA_690_ (green), PA_825_ (red), and ratio PA_690_/PA_825_ were obtained. Reproduced with permission.^[177]^ Copyright 2022, Wiley‐VCH GmbH.

H_2_S functions as a multifunctional regulator within mitochondria, implicated in various diseases stemming from mitochondrial dysfunction.^[177]^ It plays a pivotal role in numerous physiological processes, including cellular protection in the cardiovascular system, regulation of neuronal excitability, and mitigation of oxidative stress. Consequently, the development of fluorescent probes for detecting H_2_S is paramount.[^178^, ^179^, ^180^, ^181^] In comparison to FL activation methods, ratio probes with dual or multiple emissions establish self‐calibration systems that mitigate environmental influences such as probe concentration and excitation intensity. To precisely diagnose and treat H_2_S‐related disorders, Shi et al. devised a ratio‐based NP for quantitative imaging and clearance of endogenous H_2_S.^[182]^ They synthesized ZM1068‐NB, a near‐infrared fluorescent probe, wherein the 4‐nitrobenzoate moiety underwent spontaneous ketone enol copolymerization with H_2_S to yield ZM1068‐Ketone (Figure 13a). Upon reacting with H_2_S, the absorption intensity of ZM1068‐NB gradually diminished at 900 nm while increasing at 680 nm, rendering it suitable for ratio PAI (PA_680_/PA_900_). Furthermore, its emission wavelength shifted from 1070 nm pre‐reaction to 720 nm post‐reaction. Ultimately, the molecule's ability to generate ROS was activated post‐reaction, affording it PDT capabilities. To enhance the water solubility and targeting capability of ZM1068‐NB, mPEG5000‐PCL3000‐FA was used to encapsulate it, forming ZNNPs@FA. At the cellular level, ZNNPs@FA effectively targeted HCT116 cells and responded to endogenous H_2_S for cellular imaging, with the generated ROS inhibiting cancer cell growth. Given the significant association between H_2_S and various liver diseases as well as its impact on the central nervous system, the H_2_S levels in the livers and brains of mice with brain injury were evaluated based on the aforementioned research findings. FL signals at 720 and 1070 nm were notably observed in the diseased areas of the experimental group, with FL intensity increasing gradually over time, indicating substantial probe aggregation in these regions (Figure 13b). The experimental group exhibited the highest signal intensity in PA_680_, while PA_900_ showed the weakest signal (Figure 13c). Additionally, a BALB/c mouse model with HCT116 tumors was established, demonstrating that the experimental group effectively suppressed tumor growth through H_2_S consumption and PDT activation in combination therapy. This molecule has paved the way for specific imaging and treatment of H_2_S‐related diseases.

H_2_S is not only highly expressed in tumor sites but also in liver inflammation. This can be used for exploring the pathological processes related to redox imbalance in the liver. Ye et al. reported an electron color changing molecule with reversible response to ^•^OH and H_2_S.^[177]^ Due to the changes in π electronic structure within the molecule, it exhibited different NIR absorption between redox states, making it suitable for reversible PAI probes. They designed and synthesized a small molecule EM1 based on an organic π‐electron structure, which can be rapidly oxidized by H_2_S to EM1^2+^ (an oxidative double cation mode). The product color was violet. When ^•^OH was added, EM1^2+^ was reduced to the structure of EM1 (a reducing diene form), which had bright yellow color. Ratio probes can improve imaging accuracy through self‐calibration effects, so they mixed EM1 molecules with a NIR semiconductor polymer [2,6‐(4,4‐bis‐(2‐ethylhexyl)‐4H‐cyclopenta[2,1‐b; 3,4‐b] dithiophene)‐alt‐4.7(2,1,3‐benzothiadiazole)] (PCPDTBT) that had stable absorption at 825 nm to form nanoparticles (1‐PAIN) (Figure 13d). Because EM1 had a weak absorption peak at 690 nm, while PCPDTBT had a strong absorption peak at 825 nm, the PA of 1‐PAIN was relatively low between 690 and 825 nm. After the addition of ^•^OH, EM1 was rapidly oxidized to EM1^2+^, with a significant increase in absorption at 690 nm, while the signal at 825 nm remained unchanged before and after the response. Therefore, the PA of 1^2+^‐PAIN was significantly enhanced between 690 and 825 nm. After adding H_2_S, it would be oxidized again and enter the 1‐PAIN state, while the ratio of PA_690_/PA_825_ returned to its initial state (Figure 13e). Then, at the cellular and animal levels, the endogenous ^•^OH and H_2_S oxidation cycle in mouse liver was monitored using 1‐PAIN. Lipopolysaccharide (LPS) was injected into mice to induce liver tissue inflammation, resulting in an increase in ^•^OH content in the liver area, a significant enhancement of PA_690_ signal, and gradual brightening of the PA_690_/PA_825_ ratio image. Subsequently, injection of *N*‐acetyl cysteine (NAC) induced anti‐inflammation process increased the production of H_2_S in the liver. The PA_690_ signal in the liver gradually weakened, the PA_825_ signal did not show significant changes, and the ΔPA_690_/ΔPA_825_ ratio decreased. These imaging results were consistent with the results of the experimental group after intraperitoneal injection of *L*‐Cysteine (*L*‐Cys) in mice treated with LPS (Figure 13f). This probe can accurately monitor real‐time information on the redox status in the body, which provides a very advantageous imaging tool for the study of redox imbalance diseases.

## 8 CONCLUSION AND PERSPECTIVES

The Jablonski diagram functions as the basic photophysical principle for controlling phototheranostic performance. The regulation of excited state energy transformation processes is effective to optimize the disease diagnosis and therapy outcomes. The stimuli‐responsive or activatable molecular probes have attracted tremendous attention as they hold great potential for precision medicine. In this review, we summarize the recent advances of activatable molecular probes of which the photophysical energy transformation process can be tuned to make the excited state energy concentrated on a certain dissipation pathway for smart disease theranostics. We discuss the external stimuli‐triggered molecular structure changes that result in desirable photophysical properties. We also highlight the assembly and disassembly of molecular aggregates that greatly affect the photophysical energy transformation processes. The biomedical applications of these smart molecular probes in serious diseases such as cancer and inflammation are also illustrated.

Although activatable molecular probes have made significant progress in regulating photophysical energy conversion processes, several challenges remain to be addressed. Firstly, as noted in the literature we have reviewed, the vast majority of disease models focus predominantly on tumors. While the imaging modalities have become increasingly diversified, the treatment approaches remain relatively limited, often relying solely on PDT or PTT. Future research could explore the integration of these probes with prodrugs or proteolysis‐targeting chimeras (PROTACs), potentially leading to enhanced therapeutic outcomes. Additionally, one challenge with altering the aggregate state of materials to modulate photophysical properties is the lack of control over particle size in vivo, which could significantly affect biological metabolism. Moreover, most molecules reported in the literature rely on enhanced permeability and retention (EPR) effects for enrichment at disease sites. The use of targeted peptides and other functional modifications could significantly improve the aggregation of nanomaterials at these sites, thereby enhancing both diagnostic and therapeutic efficacy. This review aims to provide comprehensive insights into the development of more precise diagnostic and therapeutic molecular probes, thereby advancing the field of smart medicine.

## CONFLICT OF INTEREST STATEMENT

The authors declare no conflicts of interest.
